# Maximum Standardized Uptake Value of ^18^F-deoxyglucose PET Imaging Increases the Effectiveness of CT Radiomics in Differentiating Benign and Malignant Pulmonary Ground-Glass Nodules

**DOI:** 10.3389/fonc.2021.727094

**Published:** 2021-12-17

**Authors:** Rong Niu, Jianxiong Gao, Xiaoliang Shao, Jianfeng Wang, Zhenxing Jiang, Yunmei Shi, Feifei Zhang, Yuetao Wang, Xiaonan Shao

**Affiliations:** ^1^ Department of Nuclear Medicine, The Third Affiliated Hospital of Soochow University, Changzhou, China; ^2^ Changzhou Key Laboratory of Molecular Imaging, Changzhou, China; ^3^ Department of Radiology, the Third Affiliated Hospital of Soochow University, Changzhou, China

**Keywords:** lung adenocarcinoma, radiomics, standardized uptake value, fluorodeoxyglucose F18, positron emission tomography-computed tomography

## Abstract

To investigate whether the maximum standardized uptake value (SUVmax) of ^18^F-deoxyglucose (FDG) PET imaging can increase the diagnostic efficiency of CT radiomics-based prediction model in differentiating benign and malignant pulmonary ground-glass nodules (GGNs). We retrospectively collected 190 GGNs from 165 patients who underwent ^18^F-FDG PET/CT examination from January 2012 to March 2020. Propensity score matching (PSM) was performed to select GGNs with similar baseline characteristics. LIFEx software was used to extract 49 CT radiomic features, and the least absolute shrinkage and selection operator (LASSO) algorithm was used to select parameters and establish the Rad-score. Logistic regression analysis was performed combined with semantic features to construct a CT radiomics model, which was combined with SUVmax to establish the PET + CT radiomics model. Receiver operating characteristic (ROC) was used to compare the diagnostic efficacy of different models. After PSM at 1:4, 190 GGNs were divided into benign group (n = 23) and adenocarcinoma group (n = 92). After texture analysis, the Rad-score with three CT texture features was constructed for each nodule. Compared with the Rad-score and CT radiomics model (AUC: 0.704 (95%CI: 0.562-0.845) and 0.908 (95%CI: 0.842-0.975), respectively), PET + CT radiomics model had the best diagnostic efficiency (AUC: 0.940, 95%CI: 0.889-0.990), and there was significant difference between each two of them (*P* = 0.001-0.030). SUVmax can effectively improve CT radiomics model performance in the differential diagnosis of benign and malignant GGNs. PET + CT radiomics might become a noninvasive and reliable method for differentiating of GGNs.

## Introduction

Lung cancer is the leading cause of cancer-related deaths worldwide, especially in China (1, 2). The incidence of lung cancer is increasing rapidly. It is predicted that China’s lung cancer mortality will increase by about 40% from 2015 to 2030 (1, 2). Early diagnosis and treatment are crucial for improving the prognosis of patients. With the significant increase in the detection of many asymptomatic pulmonary nodules and the change in the epidemiological trend of lung cancer in China, diagnosis, and differentiation of ground-glass pulmonary nodules (GGNs) has become a huge challenge for clinicians (3). It is reported that the probability of malignancy of GGNs is higher than that of solid pulmonary nodules (4), but it can also be caused by benign lesions such as organizing pneumonia and interstitial pneumonia. High-resolution computed tomography (HRCT) is generally recognized as a routine method for differentiating GGNs. However, the radiological features of benign and malignant GGNs are overlapping, and the judgment of the characteristics of the lesion is easily to be affected by subjective factors. Therefore, the diagnostic efficiency of HRCT needs to be improved (5). According to the recommendation of guidelines for Management of Incidental Pulmonary Nodules Detected on CT Images, pulmonary GGNs that cannot be characterized can be further identified by CT follow-up to observe the dynamic changes of GGNs (6). However, some benign GGNs and early lung adenocarcinoma remain stable for a long time, making it difficult to differentiate them (7). Moreover, long-term follow-up often brings panic and anxiety to patients. Pathological examination is the gold standard for the diagnosis of GGNs. However, the cell composition of GGN is relatively small, which requires a highly skillful puncture technique for pathological examination that is difficult to perform. Thus, bronchoscopy and percutaneous lung puncture techniques have limited application value in GGNs. Therefore, it is urgent to develop a reliable and practical noninvasive imaging method to accurately distinguish benign and malignant GGNs to guide the individualized clinical management strategy for GGNs.

Radiomics is a very promising diagnostic method. With the help of mathematical and statistical methods, high-throughput characteristic spatial data can be extracted from the image data of the region of interest, and valuable lesion information that the naked eye may ignore can be effectively captured to improve the accuracy of disease diagnosis (8–10). Radiomics has the advantages of real-time, objective, noninvasive, and reusability. Previous studies have shown that CT texture features are potentially radiological biomarkers in the differential diagnosis of lung cancer (11, 12), the prediction of tumor growth (13), gene expression (14), and the evaluation of therapeutic efficacy (9, 15). Most of these studies are based on solid pulmonary nodules, and there are few reports on the differentiation of benign and malignant GGNs. Theoretically, inflammatory lesions and malignant tumors have completely different biological behaviors, pathological processes, and internal spatial structures. Therefore, radiomics that is based on multi-dimensional characteristics can identify malignant lesions from benign GGNs. Digumarthy et al. (16) differentiated benign and malignant lesions in 108 GGNs obtained from 36 patients and found that only 2/92 radiomic features (cluster shade and surface volume ratio) could be used for model prediction, with AUC=0.624, which is of diagnostic value, but its diagnostic efficacy needs to be improved. It has been reported that the model with the combination of radiomics and semantic parameters can improve the performance of radiomics model alone (17). Besides, PET/CT imaging, as noninvasive dual-modality imaging that reflects tumor heterogeneity, has been recognized for its application in the field of lung cancer. Our previous studies also found that PET metabolic parameters help identify GGNs (18). Thus, we proposed that adding PET metabolic parameters (SUV) on the basis of CT radiomics model will be beneficial in the differentiation of benign and malignant GGNs.

The purpose of this study was to establish a dual-modality comprehensive prediction model based on CT texture parameters, semantic features, and PET metabolic parameters through analyzing the PET/CT images of patients with indeterminate lung GGNs who underwent ^18^F-FDG PET/CT examination before operation and to investigate whether the SUVmax can increase the diagnostic efficiency of CT radiomics-based prediction model in differentiating benign and malignant GGNs.

## Materials and Methods

### Research Objects

This was a single-center case-control study. We retrospectively selected patients who received ^18^F-FDG PET/CT examination in our hospital from January 2012 to March 2020 for indeterminate GGNs. The study was approved by the Institutional Ethics Committee, and no informed consent was required from the patients for retrospective study [approval No.: (2020) Science No. 075]. Inclusion criteria: patients with lung GGN; GGN ≤3 cm; patients who underwent PET/CT scan and breath-holding chest CT scan in our department; the lesions were resected within 1 month after PET/CT examination, and lung adenocarcinoma and benign lesions with complete postoperative pathological data. Exclusion criteria: lesions with poor quality images that affected the measurement; patients who received any anti-tumor treatment; lung cancer patients with stage IB or above; patients with fasting blood glucose level >11.1 mmol/L; patients with impaired liver function (serum alanine aminotransferase or aspartate aminotransferase exceeding five times the upper limit of the normal value).

**Figure 1:**
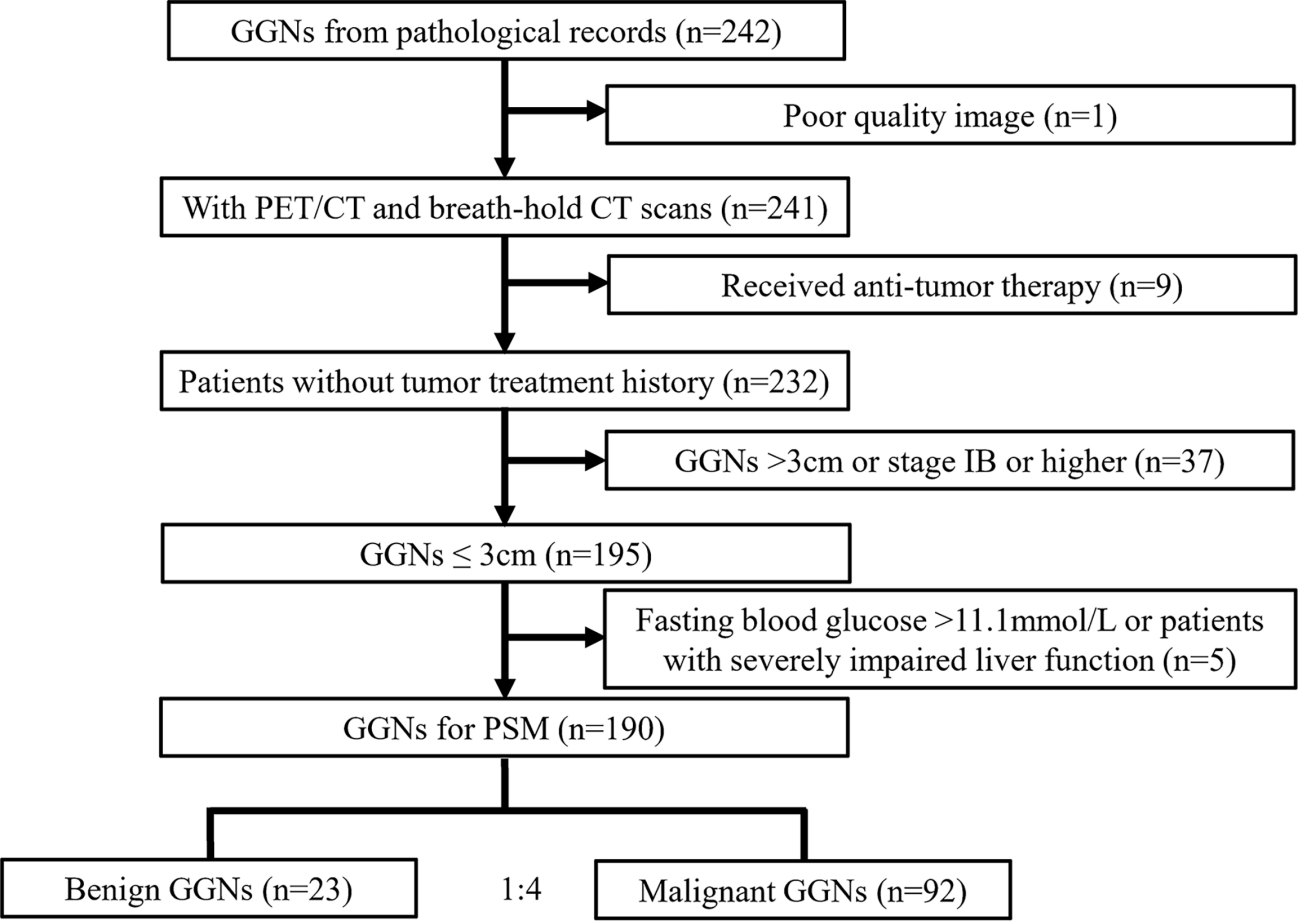
Flow chart of the study. GGN = ground-glass nodule, PSM = propensity score matching.

The total number of GGNs that met the inclusion criteria was 190, belonging to 165 patients, including 53 males and 112 females, aged 60.8 ± 9.2 years (range 31-81 years). All GGNs were divided into a benign group (n = 23) and an adenocarcinoma group (n = 167) according to postoperative pathology [the pathological classification of adenocarcinoma group was based on the classification of lung adenocarcinoma published by IASLC/ATS/ERS in 2011 (19), and the staging of the lesion was based on the Eighth Edition of the TNM Classification of Lung Cancer published by the Union for International Cancer Control (UICC) in 2017 (20)]. The patient selection process is shown in **Figure 1**.

### PET/CT Examination

PET/CT imaging was performed using Siemens biograph mCT (64) PET/CT scanner. The examination procedure was as follows: After fasting for 4-6 h, the height and weight of the patients were measured, and fingertip blood was collected for the blood glucose test. The imaging agent ^18^F-FDG was injected intravenously into the back of the hand or elbow at a dose of (3.70-5.55) MBq/kg, and the images were collected after the patient rested of (60 ± 5) min. The patient was lying on the examination table supine, with both hands holding the head. Low-dose whole-body CT scanning was performed first from skull base to middle femur, and then whole-body PET scanning was applied with the same range at 2 min/bed. Each patient was scanned for about 6-7 beds. No respiratory gating device was used during image acquisition. Image reconstruction: TrueX + TOF (ultraHD-PET) with 2 iterations and 21 subsets, and Gaussian filtering with a full-width at half maximum of 2.0 mm; matrix (pixels) 200 × 200, zoom 1.00, the image acquisition mode was 3D. The image was evaluated using TrueD software (Siemens). CT data were used for attenuation correction of the PET images, and the corrected PET images were merged with CT images. TrueD software (Siemens) was used to display and analyze the images.

### CT Examination and Image Reconstruction

After the whole-body PET/CT scan, the breath-holding chest CT scan was performed immediately, and the GGN site was reconstructed with thin slices. Acquisition and reconstruction conditions: The tube current was automatically adjusted according to the human body’s anatomical structure and tissue density. The tube voltage was 140 kV, the rotation time was 0.5 seconds, the pitch was 0.6, the kernel was B70f very sharp, the matrix was 512 × 512, the reconstruction layer thickness was 3.0 mm and 1.0 mm, the window width was 1200 HU, and the window level was -600 HU.

### Clinical and Imaging Parameters

The clinical data collected included age, sex, smoking history, and fasting blood glucose level of the patients. The CT parameters included the number of GGN (solitary, multifocal), type of GGN (pGGN, mGGN), location (central, peripheral), shape (round/oval, irregular), margin (smooth, lobulated), abnormal bronchus sign, vacuole sign, pleural indentation sign, vascular convergence sign. PET parameter: SUVmax of nodules.

### Texture Analysis and Feature Extraction

The patient’s chest CT images were exported from Siemens workstation in DICOM format and uploaded into LIFEx software (version 5.10, http://www.lifexsoft.org). Two experienced nuclear medicine doctors (Niu R and Shao X) manually delineated each target lesion, drew the region of interest (ROI) layer by layer along the lesion’s contour, and automatically calculated and extracted texture features for each GGN.

### Statistical Analysis

First, the GGNs that met the inclusion criteria were matched with propensity score (PSM) according to benign to malignant ratio at 1:4. The PS variables were age, sex, smoking history, fasting blood glucose level, and GGN number grouping (**Supplementary Table S1**). The continuous variables with normal distribution were expressed as the mean ± standard deviation (SD), continuous variables with non-normal distribution were expressed as the median (Q1-Q3), and the categorical variables were expressed as frequency (%). T-test or Mann Whitney U test was used to compare continuous variable data between groups, chi-square test or Fisher test was used to compare categorical data between groups.

For texture feature selection, we first removed two parameters with intraclass correlation coefficients (ICCs) lower than 0.75. Next, the Mann Whitney U test was performed to screen the parameters between benign and adenocarcinoma groups (*P*-value was relaxed to 0.15). The best predictive features were selected using the least absolute shrinkage and selection operator (LASSO) algorithm and 10-fold cross-validation, and the Rad-score of each GGN was calculated. Multivariable logistic regression analysis was carried out to construct the joint model using Rad-score, CT conventional morphological parameters (semantic features), and PET parameters (SUVmax) with different CT reconstruction slice thickness. The receiver operating characteristic (ROC) curve was prepared for each model, and the area under the curve (AUC) was calculated. The bootstrap resampling method (times = 500) (21) recommended by the TRIPOD statement was used to internally verify the model and calculate 95% confidence interval (CI) of AUC. Delong test was used to compare whether the differences in effectiveness between the models were statistically significant (*P <*0.05), and the nomogram of the model was generated. All statistical analyses were performed using R software, version 3.4.3 (http://www.R-project.org; software package: glmnet, pROC, rms, dca.R).

## Results

### General Data

After PSM at a ratio of 1:4, 190 GGNs were divided into benign and adenocarcinoma groups. There were 23 GGNs in benign group (including 3 organizing pneumonia, 4 fungal infections, 1 interstitial pneumonia, 5 granulomatous inflammation, and 10 other benign lesions), and 92 GGNs in adenocarcinoma group (including 77 invasive lung adenocarcinoma, 8 microinvasive adenocarcinoma, and 7 preinvasive lesions). The general data of GGNs before and after PSM is shown in **Table 1**. Comparing CT semantic features and SUVmax of GGNs after PSM, we found that only pleural indentation and SUVmax were significantly different between the benign and adenocarcinoma groups (*P <*0.001 and 0.024, respectively). There were no significant differences in nodule type, location, shape, margin, abnormal bronchus sign, vacuole sign, and vascular convergence between the benign group and adenocarcinoma group (all *P >*0.05) (**Table 2**).

**Table 1:** General data of GGNs in benign group and adenocarcinoma group before and after PSM.

	Before matching			After matching		
	Benign (n = 23)	Adenocarcinoma (n = 167)	*P*-value	Benign (n = 23)	Adenocarcinoma (n = 92)	*P*-value
Age (years)	55.8 ± 10.5	60.8 ± 8.7	0.013	55.8 ± 10.5	57.4 ± 8.9	0.477
Sex			0.002			0.428
Female	9 (39.1%)	120 (71.9%)		9 (39.1%)	47 (51.1)	
Male	14 (60.9%)	47 (28.1%)		14 (60.9%)	45 (48.9)	
History of smoking			0.039			0.800
No	15 (65.2%)	139 (83.2%)		15 (65.2%)	65 (70.7)	
Yes	8 (34.8%)	28 (16.8%)		8 (34.8%)	27 (29.3)	
Fasting blood glucose (mmol/L)	6.8 ± 1.9	6.7 ± 1.7	0.960	6.8 ± 1.9	6.65 ± 1.69	0.772
GGN number grouping			0.448			1.000
Solitary	15 (65.2%)	95 (56.9%)		15 (65.2%)	58 (63)	
Multifocal	8 (34.8%)	72 (43.1%)		8 (34.8%)	34 (37)	

Results in the table: Mean ± SD/N (%).

**Table 2:** Comparison of CT semantic features and SUVmax of GGNs between benign group and adenocarcinoma group after PSM.

Features	Benign (n = 23)	Adenocarcinoma (n = 92)	*P*-value
Type			0.625
pGGN	7 (30.4%)	33 (35.9%)	
mGGN	16 (69.6%)	59 (64.1%)	
Location			1.000
Peripheral	22 (95.7%)	88 (95.7%)	
Central	1 (4.3%)	4 (4.3%)	
Shape			0.922
Round/oval	15 (65.2%)	59 (64.1%)	
Irregular	8 (34.8%)	33 (35.9%)	
Margin			0.111
Smooth	16 (69.6%)	47 (51.1%)	
Lobulated	7 (30.4%)	45 (48.9%)	
Abnormal bronchus sign			0.080
No	10 (43.5%)	23 (25.0%)	
Yes	13 (56.5%)	69 (75.0%)	
Vacuole sign			0.904
No	19 (82.6%)	75 (81.5%)	
Yes	4 (17.4%)	17 (18.5%)	
Pleural indentation			<0.001
No	19 (82.6%)	40 (43.5%)	
Yes	4 (17.4%)	52 (56.5%)	
Vascular convergence			0.559
No	2 (8.7%)	5 (5.4%)	
Yes	21 (91.3%)	87 (94.6%)	
SUVmax	2.9 (1.4-6.9)	1.8 (1.1-3.0)	0.024

Results in the table: Median (Q1-Q3)/N (%).

### CT Texture Features Analysis

Through texture analysis, 49 features were obtained for each GGN (**Supplementary Table S2**). According to ICC analysis of two different readers, the ICCs of three texture features of 3 mm slice thickness CT images were lower than 0.75, including GLZLM_SZE, GLZLM_SZLGE, and GLZLM_ZP, indicating that these three texture features needed to be eliminated in subsequent analysis. In contrast, the ICCs of GGN texture features of 1 mm slice thickness CT images were all above 0.75 (**Supplementary Table S3**).

### Comparison of CT Texture Features of Different Slice Thickness Between Benign Group and Adenocarcinoma Group

Comparing CT texture features of different layer thicknesses of GGNs between benign group and adenocarcinoma group, we found that there were 6 parameters (CONVENTIONAL_HUmin, GLRLM_LGRE, GLRLM_SRLGE, GLRLM_LRLGE, GLZLM_LGZE, and GLZLM_LZLGE) in the texture features of 3 mm slice thickness CT images that passed the primary screening (*P* = 0.064-0.149). There were 12 parameters in the texture features of 1 mm slice thickness CT images that passed the primary screening (*P* = 0.018-0.112), including HISTO_Kurtosis, HISTO_ExcessKurtosis, HISTO_Energy, GLCM_Homogeneity, GLRLM_SRE, GLRLM_LRE, GLRLM_LRLGE, GLRLM_RP, NGLDM_Contrast, GLZLM_SZE, GLZLM_LZE, and GLZLM_ZP (**Supplementary Table S4**).

### Rad-Scores of CT Images With Different Slice Thickness

LASSO algorithm and 10-fold cross-validation were carried out to extract the best subset of CT radiomics features. For CT images of 3 mm slice thickness: Rad-score (3 mm) = −0.00198 × CONVENTIONAL_HUmin + 10.16317× GLRLM_LGRE + 66.97979 × GLRLM_SRLGE.

For CT images of 1 mm slice thickness: Rad-score (1 mm) = 11.21344 × GLRLM_LRE + 10.43443 × GLRLM_LRLGE - 0.7472 × NGLDM_Contrast.

ROC curve analysis showed that the AUC values of the Rad-score of the two reconstruction slices with different thickness were 0.634 (95%CI: 0.499-0.768) and 0.704 (95%CI: 0.562-0.845), respectively, with no significant difference between them (Z = 0.702, *P* = 0.483) (**Supplementary Figures S1**, **S2**). The diagnostic efficiency of the Rad-score of the two reconstruction slices is shown in **Table 3**.

**Table 3:** Comparison of diagnostic efficiency of different models.

Model	AUC (95%CI)	Best threshold	Sensitivity	Specificity	Accuracy
Rad-score (3 mm)	0.634 (0.499-0.768)	2.162	0.707	0.609	0.687
Rad-score (1 mm)	0.704 (0.562-0.845)	10.920	0.852	0.609	0.779
CT radiomics model (3 mm)	0.794 (0.704-0.884)	0.903	0.794	0.739	0.783
CT radiomics model (1 mm)	0.908 (0.842-0.975)	1.242	0.815	0.956	0.857
PET + CT radiomics model	0.940 (0.889-0.990)	1.931	0.815	1.000	0.870

### Rad-Score in Combination With CT Semantic Features to Construct CT Radiomics Model

**Figure 2:**
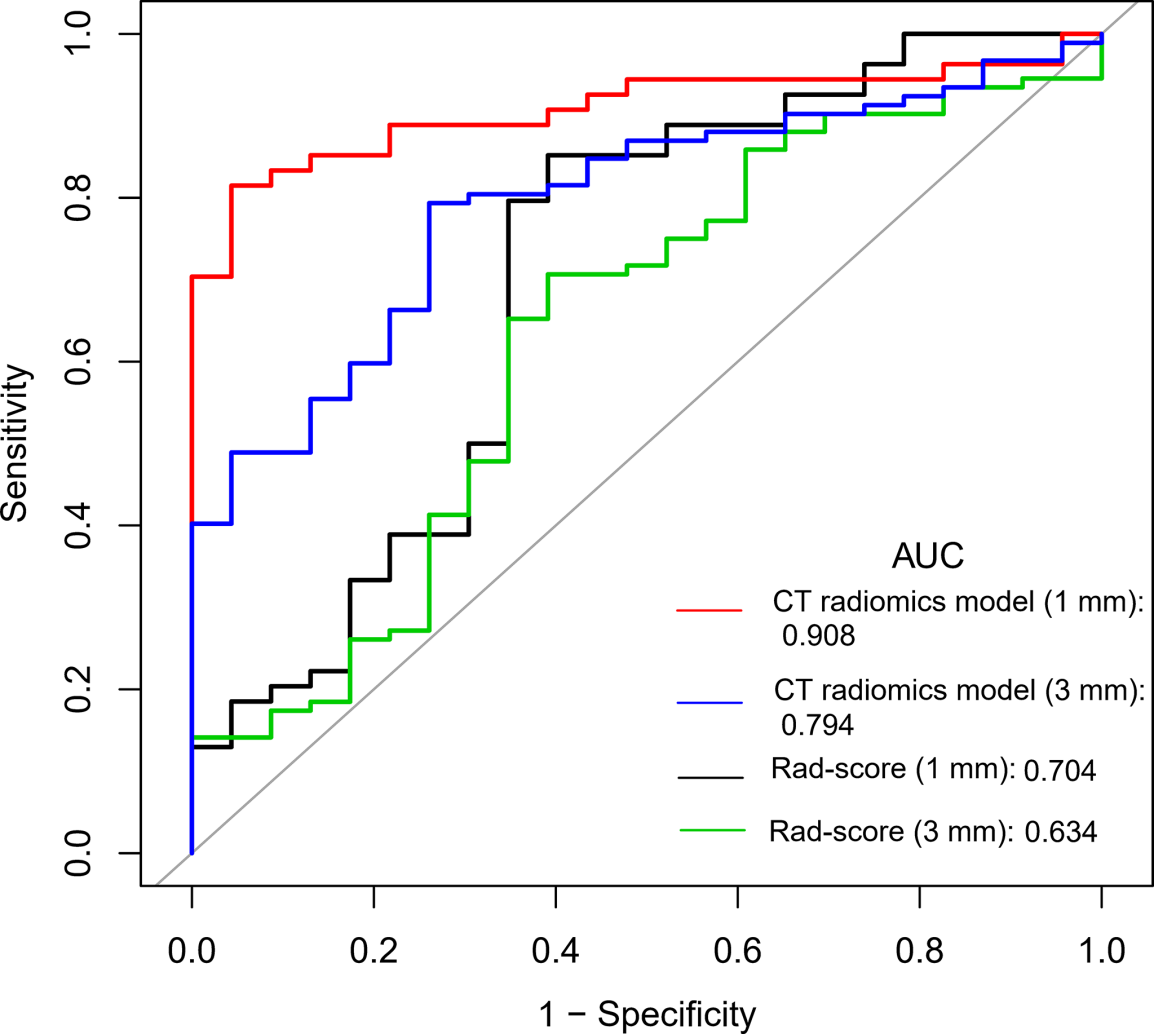
Comparison of ROC curves between Rad-score and radiomics models with different CT reconstruction slice thickness.

Multivariable logistic regression analysis was performed to construct the CT radiomics models to predict the benign and malignant GGNs using semantic characteristic parameters of GGNs and the Rad-scores of CT with different slice thickness individually. The formula of CT radiomics model (3 mm) was as follows: CT radiomics model (3 mm) = −5.20333 + 1.16220 × (abnormal bronchus sign = 1) + 2.12571 × (pleural indentation sign =1) + 2.23666 × Rad-score (3 mm). The AUC value of the 3 mm CT radiomics model was 0.794 (95%CI: 0.704-0.884), which was significantly higher than that of Rad-score (3 mm) (Z = 2.232, *P* = 0.026) (**Figure 2**).

The formula of CT radiomics model (1 mm) was as follows: CT radiomics model (1 mm) = −45.94109 + 3.06092 × (abnormal bronchus sign = 1) + 3.33342 × (pleural indentation sign = 1) + 3.89642 × Rad-score (1 mm). The AUC value of the model was 0.908 (95%CI: 0.842-0.975), which was significantly higher than that of Rad-score (1 mm) (0.704) (Z = 2.769, *P* = 0.006) and CT radiomics model (3 mm) (0.794) (Z = 1.998, *P* = 0.046) (**Figure 2**). The diagnostic efficiency of different models is shown in **Table 3**.

### Construction of PET + CT Radiomics Model

**Figure 3:**
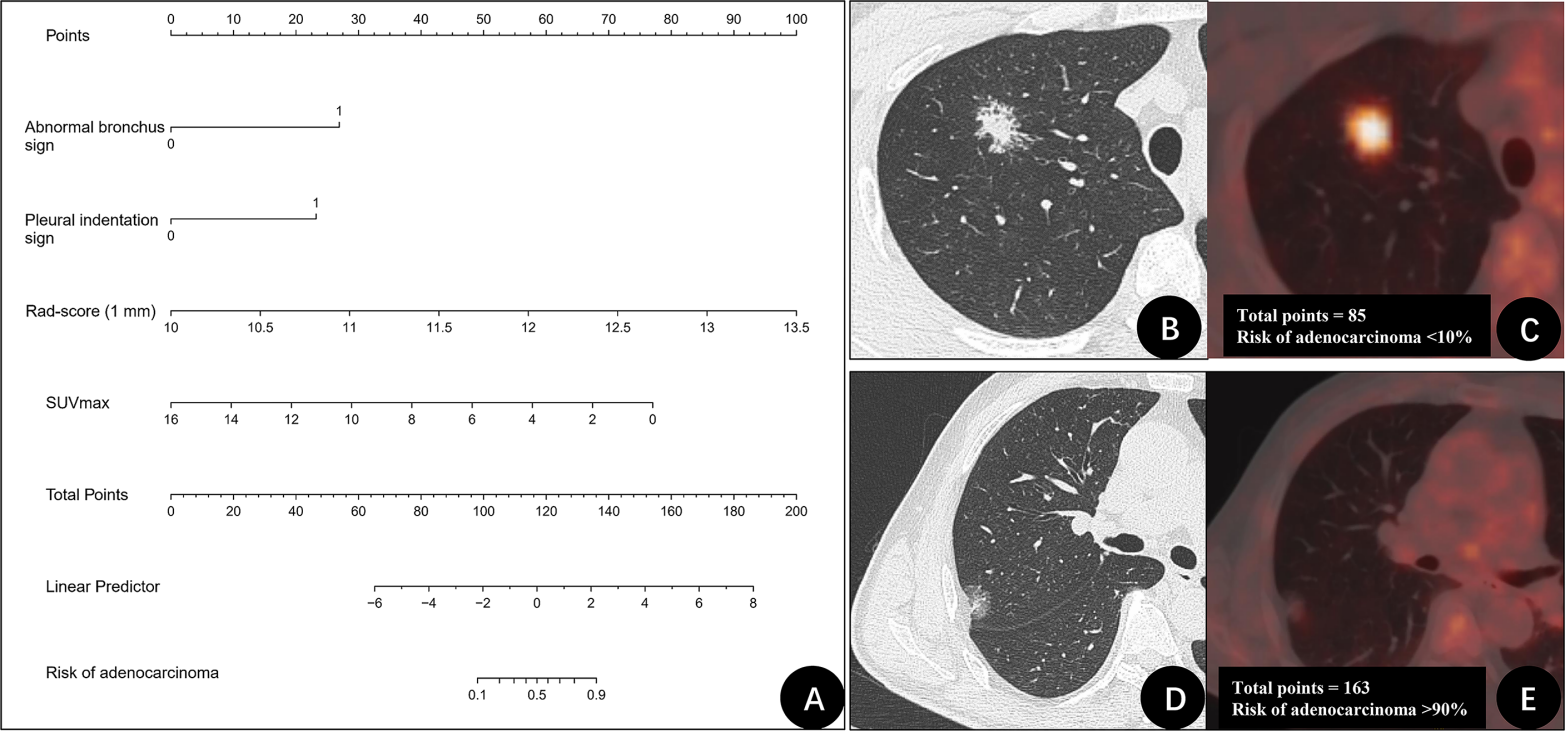
The nomogram of PET + CT radiomics model for differentiating benign and malignant GGNs and two examples. **(A)** Nomogram of PET + CT radiomics model. **(B, C)** A 31-year-old man with a ground-glass nodule (GGN) on the right upper lung lobe. CT image **(B)** and PET/CT fusion image **(C)** show that nodule with abnormal bronchus sign (27 points), and no pleural indentation was identified (0 points). Rad-score (1 mm) was 10.7 (20 points), maximum standardized uptake value (SUVmax) was 8.1 (38 points). The total points were 85 points. The risk of adenocarcinoma for this nodule was < 10%. Postoperative pathologic findings indicated granuloma. **(D, E)** A 61-year-old man with GGN on the right upper lung lobe. CT image **(D)** and PET/CT fusion image **(E)** show that nodule with abnormal bronchus sign (27 points) and pleural indentation sign (23 points). Rad-score (1 mm) was 11.3 (38 points), SUVmax was 1.1 (71 points). The total points were 163 points. The risk of adenocarcinoma for this nodule was > 90%. Postoperative pathologic findings indicated invasive adenocarcinoma.

Furthermore, based on the CT radiomics model (1 mm) and combined with SUVmax in PET parameters, a dual-modality prediction model (PET + CT radiomics model) was established to predict the benign and malignant GGNs. The formula was as follows: PET + CT radiomics model = −89.87509 + 7.01593 × (abnormal bronchus sign = 1) + 5.03616 × (pleural indentation sign = 1) +7.74753 × Rad-score (1 mm) − 0.84485 × SUVmax. The AUC of this radiomics model was 0.940 (95%CI: 0.889-0.990), and the nomogram of the model as shown in **Figure 3A**. Two examples of how to use the nomogram were showed in **Figures 3B–E**.

**Figure 4:**
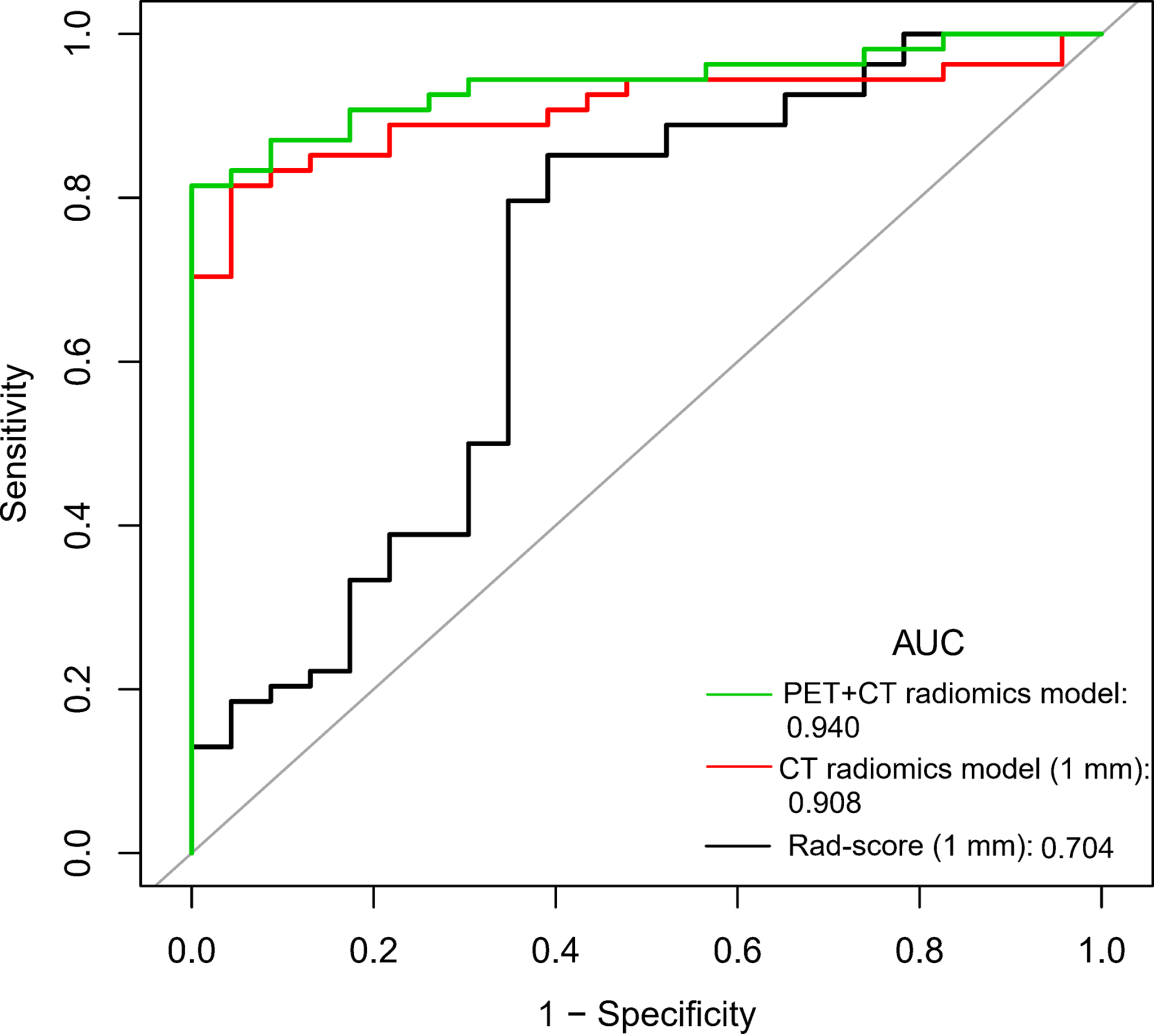
Comparison of ROC curves of Rad-score, CT radiomics model, and PET + CT radiomics model of 1 mm CT.

We compared the diagnostic efficiency of Rad-score of 1 mm CT, CT radiomics model, and PET + CT radiomics model in differentiating benign and malignant GGNs. The results showed that the AUC values of the three models were significantly different from each other, with Z = 2.174-3.304 and *P* = 0.001-0.030, and the PET + CT radiomics model had the highest diagnostic efficiency (**Figure 4**). The comparison of diagnostic efficiency of different models is shown in **Table 3**.

## Discussion

This study systematically analyzed the semantic features of suspected lung GGNs and 49 radiomics features extracted from each GGN and constructed a CT radiomics model with abnormal bronchus sign, pleural indentation sign, and Rad-score. This CT radiomics model had good differentiation efficiency in benign and malignant GGNs. We further added SUVmax to the CT radiomics model to construct the PET + CT radiomics model, which effectively improved the predictive value of CT radiomics model in differentiating benign and malignant GGNs.

After PSM, the proportion of pleural indentation sign in CT semantic features of adenocarcinoma group was significantly higher than that of benign GGNs. Pleural indentation sign refers to the pleural indentation caused by the traction of subpleural lesions on adjacent pleura, which mostly presents as linear or triangular shadow. Hu et al. reported that this sign is one of the common features of peripheral lung cancer (22). Although some semantic features play an important role in clinical application, they depend on the experience of radiologists or surgeons and their understanding of the signs, and thus they are easily affected by subjective factors. Previous literature also reported that single CT parameters, such as nodule margin characteristics, size, and CT value, have limited values in differentiating benign and malignant GGNs (23). Besides, in univariate comparison, we found that SUVmax of benign GGNs was higher than that of malignant GGNs, which is consistent with the findings of McDermott et al. (24) and Chun et al. (25). This may be because benign GGN is more common in tuberculosis, organizing pneumonia, and other inflammatory lesions. It is well known that SUV is a semi-quantitative parameter that reflects the uptake of ^18^F⁃FDG in the lesion. The increase of SUV value represents the increased uptake of glucose in the lesion, the strong proliferation and growth of cells, and the possibility of malignant tumor. However, ^18^F-FDG is not a tumor-specific imaging agent. The chemotaxis of inflammatory factors and the accumulation of inflammatory cells may also cause a significant increase in the uptake of FDG (26, 27). On the contrary, early lung adenocarcinoma manifesting as GGN showed lower density, slower growth, less expression of glucose transporter-1 (GLUT-1), less uptake of FDG, and lower SUVmax (28, 29).

This study found that the consistency of CT images 1 mm slice is better, whereas there are three parameters with greater variability in 3 mm CT texture features (including GLZLM_SZE GLZLM_SZLGE, and GLZLM_ZP). In the analysis of more than 100 PET texture features, Leijenaar et al. (30) also found that GLZLM feature-based parameters have the highest variability. In our study, we also found that the number and types of texture features of CT images that were significantly different between benign group and adenocarcinoma group were different with different slice thickness, which may be related to the different spatial information display of image details on CT images with different reconstruction slice thickness. Some scholars reported that applying different reconstruction software, parameter settings, and reconstruction methods can affect radiomics features extraction (31–33).

The Rad-score (3 mm) contains the traditional CT parameters, namely, CONVENTIONAL_HUmin and two GLRLM texture features. HUmin is the minimum CT value of VOI in lesions. The lower the HUmin value is, the higher the possibility of adenocarcinoma is. This may be because malignant GGN is more prone to exhibit vacuole signs than benign lesions. GLRLM was introduced by Galloway (34), which assesses the distribution of discretized grey levels on an image or a stack of images. It describes the roughness or smoothness of the image and reflects the heterogeneity of the tumor. In addition to GLRLM, Rad-score (1 mm) parameter NGLDM_Contrast was also used in the radiomics model. NGLDM_Contrast is the intensity difference between neighboring regions, which provides information about the spatial relationship between an image voxel and its neighboring voxels (35). The lower the NGLDM_Contrast, the higher the possibility of adenocarcinoma. It has been reported that NGLDM_Contrast is also an important prognostic factor for lung cancer, and poor prognosis is associated with low NGLDM_Contrast (36). Comparing the Rad-score between slices with different reconstruction thickness, we found that although the diagnostic efficiency of Rad-score (1 mm) was slightly higher than that of Rad-score (3 mm), there was no statistical difference, and the diagnostic efficiency of both Rad-score (1 mm) and Rad-score (3 mm) was moderate, which is similar to the finding of Digumarthy (15) (AUC = 0.624).

In this study, the diagnostic efficiency of the CT radiomics model was significantly better than that of Rad-score alone, which is consistent with Hyun et al. (37) and Bianconi et al. (38). Moreover, the thin slice CT radiomics model’s prediction efficiency is better than that of the 3 mm slice CT radiomics model, which agrees with the previous reports (31). This may be because a thin-slice CT image can improve the image’s spatial resolution and facilitate the display of confidential information of lesions. In the thin slice CT radiomics model, the abnormal bronchus sign is also an independent risk factor. Bronchus sign refers to the appearance of air containing bronchus in the lesion. When bronchus is dilated, distorted or cut-off truncated, it often indicates the possibility of malignant lesions. Thus, this sign is called an abnormal bronchus sign (39). Finally, compared with Rad-score and CT radiomics model, PET + CT radiomics model had the best diagnostic efficiency (AUC = 0.940). PET imaging parameters reflect the lesions’ functional and metabolic status, provide quantitative information at the molecular level, and complement CT’s anatomical images. The establishment of the dual-modality comprehensive PET/CT model helps evaluate the lesions at multiple levels.

There are still some limitations to this study. First of all, patients enrolled in this study were those who received preoperative PET/CT differential diagnosis and staging because of suspicious GGNs, and therefore the number of benign cases is small, which is the reason why we conducted PSM. In addition, there were many types of diseases in the benign group. This heterogeneity may affect the reliability of the model. The radiomics model constructed in this study may be only suitable for the differential diagnosis of GGNs that cannot be determined on CT but not for the screening of GGNs. Secondly, our previous study showed that LIFEx software has requirements for voxels, and it is not suitable for some small or low uptake GGNs. Therefore, we did not perform a PET texture analysis. PET radiomics needs to be further explored. Thirdly, we chose the manual segmentation method for ROI delineation, which is not as stable as the fully/semi-automatic segmentation method. In a future study, we may try to use the AI-based segmentation method to obtain image information. Fourth, although the PET + CT radiomics model has good internal validation performance, external data remain needed to confirm the robustness and applicability of this radiomics model.

In conclusion, in this study, we successfully constructed CT texture feature-based Rad-score, a CT radiomics model using Rad-score in combination with semantic features (abnormal bronchus sign and pleural indentation sign) and a PET + CT radiomics model using CT radiomics model in combination with SUVmax to differentiate early lung adenocarcinoma from benign lung GGNs and compared the differential diagnostic efficacy of these models. PET + CT radiomics model has the best risk prediction performance and might become a noninvasive and reliable diagnostic tool for differentiating benign and malignant GGNs.

## Data Availability Statement

The original contributions presented in the study are included in the article/**Supplementary Material**. Further inquiries can be directed to the corresponding authors.

## Ethics Statement

Our study was approved by the ethics committee of the Third Affiliated Hospital of Soochow University for retrospective analysis and did not require informed consent. The patients/participants provided their written informed consent to participate in this study.

## Author Contributions

RN and XNS contributed to the study concepts and the study design. RN, JG, YS, FZ and ZJ contributed to data acquisition and reconstructions. XNS, RN, XLS, and ZJ contributed to data analyses and interpretation. XNS contributed to the statistical analysis. XNS, RN, and YW contributed to the manuscript preparation and manuscript editing and reviewing. All authors contributed to the article and approved the submitted version.

## Funding

Funding from Young Talent Development Plan of Changzhou Health Commission (CZQM2020012) and Key Laboratory of Changzhou High-tech Research Project (Grant No. CM20193010).

## Conflict of Interest

The authors declare that the research was conducted in the absence of any commercial or financial relationships that could be construed as a potential conflict of interest.

## Publisher’s Note

All claims expressed in this article are solely those of the authors and do not necessarily represent those of their affiliated organizations, or those of the publisher, the editors and the reviewers. Any product that may be evaluated in this article, or claim that may be made by its manufacturer, is not guaranteed or endorsed by the publisher.

## Abbreviations

18F-FDG, 18F-fluorodeoxyglucose; AUC, area under the curve; CI, confidence interval; GGN, ground-glass nodule; HRCT, High-resolution computed tomography; ICC, intraclass correlation coefficients; LASSO, least absolute shrinkage and selection operator; mGGN, mixed ground-glass nodule; PET, positron emission tomography; PSM, propensity score matching; pGGN, pure ground-glass nodule; ROC, receiver operating characteristic; ROI, region of interest; SD, standard deviation; SUVmax, maximum standardized uptake value.
