# Supplementary material for: Maximum Standardized Uptake Value of 18F-deoxyglucose PET Imaging Increases the Effectiveness of CT Radiomics in Differentiating Benign and Malignant Pulmonary Ground-Glass Nodules
Source: Front Oncol. 2021 Dec 17;11:727094. doi: 10.3389/fonc.2021.727094 (PMC8718929; doi:10.3389/fonc.2021.727094)
Supplement: Supplementary file 1 [file DataSheet_1.docx]

**Supplementary materials**

**Supplementary Table S1.** **Propensity score matching parameter table for GGNs**

| Variables to calculate the propensity score | Age, Sex, History of smoking, Fasting blood glucose, GGN number grouping |
| --- | --- |
| Propensity score algorithm | Logistic regression model |
| C-statistical | 0.7261 |
| Matching method | Greed matching within specified caliper distances |
| Distance metric | 0.7 |
| Matching ratio | 1:4 |
| Use of replacement | With replacement |
| Matching sample size | Benign GGNs (n = 23), malignant GGNs (n = 92) Total (n = 115) |

**Supplementary Table S2.** **Texture parameter features**

| **Conventional Indices** |
| --- |
| **CT** |
| HUmin  HUmean  HUstd  HUmax  HUQ1  HUQ2  HUQ3 |
| **First Order Features** |
| SHAPE_Volume (mL)  SHAPE_Volume (#vx)  SHAPE_Sphericity  SHAPE_Compacity  HISTO_Skewness  HISTO_Kurtosis  HISTO_ExcessKurtosis  HISTO_Entropy_log_10_  HISTO_Entropy_log_2_  HISTO_Energy |
| **Second Order Features** |
| **Grey Level Co-occurrence Matrix (GLCM)** |
| GLCM_Homogeneity  GLCM_Energy  GLCM_Contrast  GLCM_Correlation  GLCM_Entropy_log_10_  GLCM_Entropy_log_2_  GLCM_Dissimilarity |
| **Grey-Level Run Length Matrix (GLRLM)** |
| Short-run emphasis (SRE)  Long-run emphasis (LRE)  Low grey-level run emphasis (LGRE)  High grey-level run emphasis (HGRE)  Short-run low grey-level emphasis (SRLGE)  Short-run high grey-level emphasis (SRHGE)  Long-run low grey-level emphasis (LRLGE)  Long-run high grey-level emphasis (LRHGE)  Grey-level non-uniformity for run (GLNU)  Run length non-uniformity (RLNU)  Run percentage (RP) |
| **Neighborhood Grey-Level Different Matrix (NGLDM)** |
| NGLDM_Coarseness  NGLDM_Contrast  NGLDM_Busyness |
| **Grey-Level Zone Length Matrix (GLZLM)** |
| Short-zone emphasis (SZE)  Long-zone emphasis (LZE)  Low grey-level zone emphasis (LGZE)  High grey-level zone emphasis (HGZE)  Short-zone low grey-level emphasis (SZLGE)  Short-zone high grey-level emphasis (SZHGE)  Long-zone low grey-level emphasis (LZLGE)  Long-zone high grey-level emphasis (LZHGE)  Grey-level non-uniformity for zone (GLNU)  Zone length non-uniformity (ZLNU)  Zone percentage (ZP) |

**Supplementary Table S3. Comparison of ICC of GGNs texture parameters of CT images with different slice thickness by two readers**

| Texture parameters | 3mm | 1mm |
| --- | --- | --- |
| CONVENTIONAL_HUmin | 0.750 | 0.918 |
| CONVENTIONAL_HUmean | 0.929 | 0.957 |
| CONVENTIONAL_HUstd | 0.978 | 0.975 |
| CONVENTIONAL_HUmax | 0.981 | 0.946 |
| CONVENTIONAL_HUQ1 | 0.833 | 0.869 |
| CONVENTIONAL_HUQ2 | 0.908 | 0.944 |
| CONVENTIONAL_HUQ3 | 0.959 | 0.985 |
| SHAPE_Volume (mL) | 0.976 | 0.973 |
| SHAPE_Volume (# vx) | 0.975 | 0.969 |
| SHAPE_Sphericity | 0.904 | 0.862 |
| SHAPE_Compacity | 0.956 | 0.986 |
| HISTO_Skewness | 0.910 | 0.967 |
| HISTO_Kurtosis | 0.927 | 0.985 |
| HISTO_ExcessKurtosis | 0.927 | 0.985 |
| HISTO_Entropy_log10 | 0.986 | 0.990 |
| HISTO_Entropy_log2 | 0.986 | 0.990 |
| HISTO_Energy | 0.984 | 0.987 |
| GLCM_Homogeneity | 0.979 | 0.991 |
| GLCM_Energy | 0.973 | 0.959 |
| GLCM_Contrast | 0.978 | 0.985 |
| GLCM_Correlation | 0.929 | 0.944 |
| GLCM_Entropy_log10 | 0.977 | 0.987 |
| GLCM_Entropy_log2 | 0.977 | 0.987 |
| GLCM_Dissimilarity | 0.979 | 0.989 |
| GLRLM_SRE | 0.950 | 0.969 |
| GLRLM_LRE | 0.950 | 0.965 |
| GLRLM_LGRE | 0.840 | 0.852 |
| GLRLM_HGRE | 0.933 | 0.968 |
| GLRLM_SRLGE | 0.830 | 0.847 |
| GLRLM_SRHGE | 0.933 | 0.968 |
| GLRLM_LRLGE | 0.882 | 0.890 |
| GLRLM_LRHGE | 0.933 | 0.969 |
| GLRLM_GLNU | 0.975 | 0.956 |
| GLRLM_RLNU | 0.975 | 0.970 |
| GLRLM_RP | 0.950 | 0.966 |
| NGLDM_Coarseness | 0.978 | 0.961 |
| NGLDM_Contrast | 0.933 | 0.971 |
| NGLDM_Busyness | 0.909 | 0.915 |
| GLZLM_SZE | 0.348 | 0.959 |
| GLZLM_LZE | 0.908 | 0.995 |
| GLZLM_LGZE | 0.763 | 0.864 |
| GLZLM_HGZE | 0.924 | 0.971 |
| GLZLM_SZLGE | 0.741 | 0.876 |
| GLZLM_SZHGE | 0.924 | 0.971 |
| GLZLM_LZLGE | 0.996 | 0.995 |
| GLZLM_LZHGE | 0.921 | 0.977 |
| GLZLM_GLNU | 0.853 | 0.966 |
| GLZLM_ZLNU | 0.970 | 0.980 |
| GLZLM_ZP | 0.324 | 0.969 |

**Supplementary Table S4. Comparison of CT texture features of different slice thickness between benign group and adenocarcinoma group**

| Texture features | P-value for 3mm CT  [Benign group (n = 23) vs. Adenocarcinoma group (n = 92)] | P-value for 1mm CT  [Benign group (n = 23) vs. Adenocarcinoma group (n = 54)] |
| --- | --- | --- |
| CONVENTIONAL_HUmin | 0.065 | 0.549 |
| CONVENTIONAL_HUmean | 0.614 | 0.299 |
| CONVENTIONAL_HUstd | 0.994 | 0.236 |
| CONVENTIONAL_HUmax | 0.892 | 0.886 |
| CONVENTIONAL_HUQ1 | 0.646 | 0.742 |
| CONVENTIONAL_HUQ2 | 0.626 | 0.363 |
| CONVENTIONAL_HUQ3 | 0.613 | 0.155 |
| SHAPE_Volume (mL) | 0.789 | 0.785 |
| SHAPE_Volume (# vx) | 0.666 | 0.790 |
| SHAPE_Sphericity | 0.469 | 0.979 |
| SHAPE_Compacity | 0.308 | 0.659 |
| HISTO_Skewness | 0.711 | 0.426 |
| HISTO_Kurtosis | 0.561 | 0.070 |
| HISTO_ExcessKurtosis | 0.561 | 0.070 |
| HISTO_Entropy_log10 | 0.676 | 0.236 |
| HISTO_Entropy_log2 | 0.676 | 0.236 |
| HISTO_Energy | 0.701 | 0.085 |
| GLCM_Homogeneity | 0.504 | 0.093 |
| GLCM_Energy | 0.150 | 0.959 |
| GLCM_Contrast | 0.291 | 0.199 |
| GLCM_Correlation | 0.226 | 0.692 |
| GLCM_Entropy_log10 | 0.189 | 0.927 |
| GLCM_Entropy_log2 | 0.189 | 0.927 |
| GLCM_Dissimilarity | 0.312 | 0.152 |
| GLRLM_SRE | 0.597 | 0.056 |
| GLRLM_LRE | 0.629 | 0.046 |
| GLRLM_LGRE | 0.089 | 0.176 |
| GLRLM_HGRE | 0.686 | 0.291 |
| GLRLM_SRLGE | 0.087 | 0.208 |
| GLRLM_SRHGE | 0.685 | 0.288 |
| GLRLM_LRLGE | 0.064 | 0.112 |
| GLRLM_LRHGE | 0.686 | 0.303 |
| GLRLM_GLNU | 0.970 | 0.749 |
| GLRLM_RLNU | 0.657 | 0.842 |
| GLRLM_RP | 0.591 | 0.046 |
| NGLDM_Coarseness | 0.243 | 0.320 |
| NGLDM_Contrast | 0.157 | 0.046 |
| NGLDM_Busyness | 0.543 | 0.542 |
| GLZLM_SZE | —— | 0.112 |
| GLZLM_LZE | 0.615 | 0.018 |
| GLZLM_LGZE | 0.084 | 0.390 |
| GLZLM_HGZE | 0.676 | 0.331 |
| GLZLM_SZLGE | —— | 0.405 |
| GLZLM_SZHGE | 0.654 | 0.295 |
| GLZLM_LZLGE | 0.069 | 0.308 |
| GLZLM_LZHGE | 0.779 | 0.867 |
| GLZLM_GLNU | 0.873 | 0.972 |
| GLZLM_ZLNU | 0.613 | 0.933 |
| GLZLM_ZP | —— | 0.091 |

Note: Due to retrospective data, only 54 of the 92 GGNs in the adenocarcinoma group had CT images with a thickness of 1 mm.


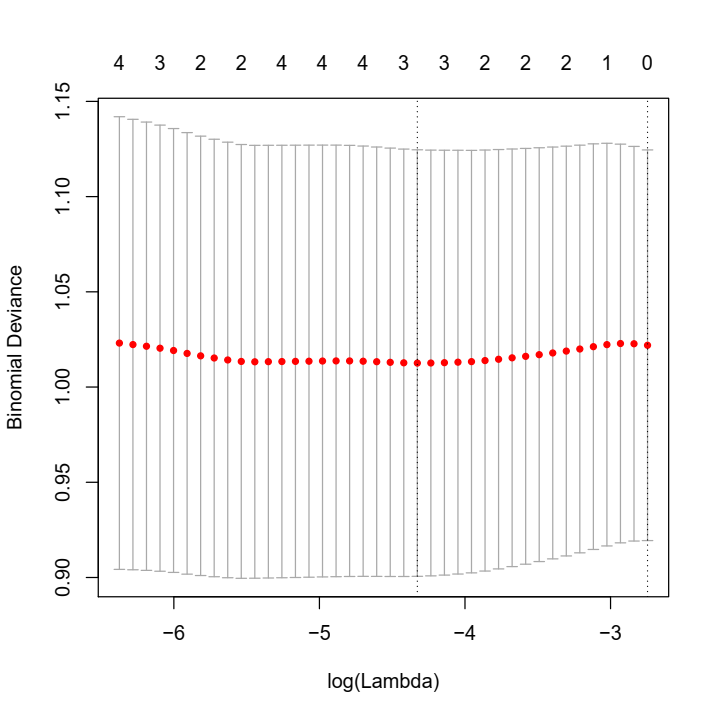

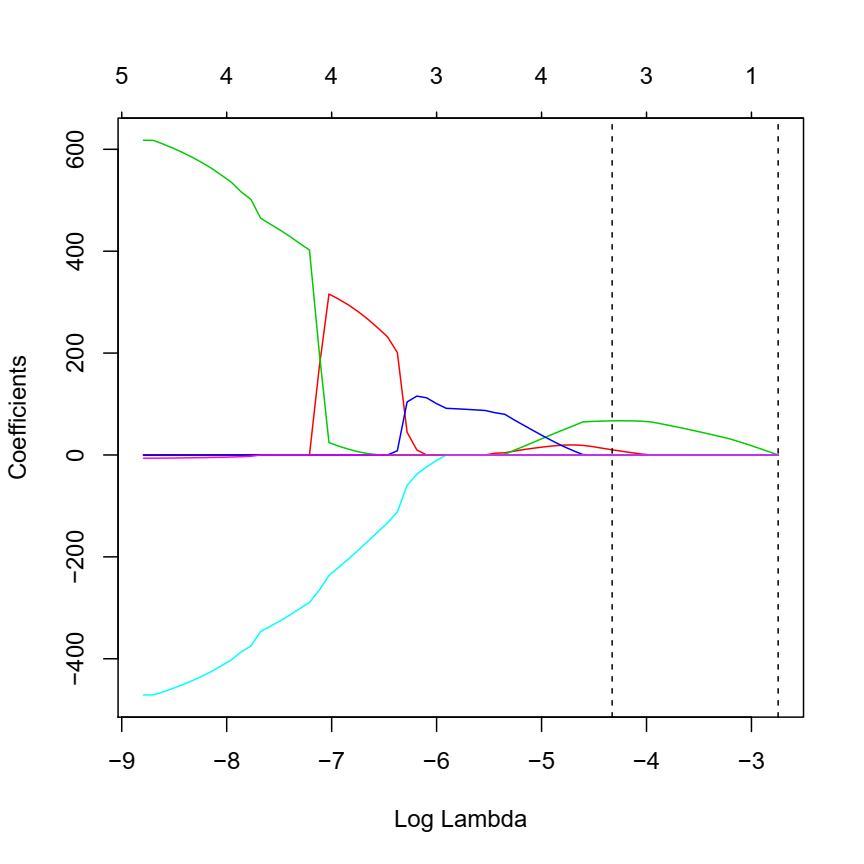

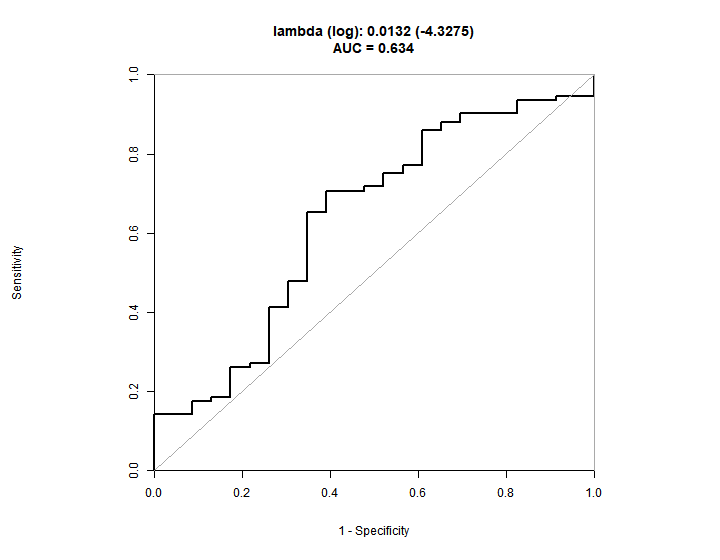


**Supplementary Figure S1.** LASSO algorithm and 10-fold cross-validation were carried out to extract the best subset of 3mm slice thickness CT radiomics features. (A) The best feature was selected based on the AUC value. The black vertical line represents the best λ value, and the model provides the best fit of the data. λ = 0.0132 with log（λ）= −4.3275 was selected as the best value. (B) LASSO coefficient curves for 6 radiomics features. The vertical line was the value selected by the 10-fold cross-validation in A, where the best λ results in 3 non-zero coefficients. (C) ROC curve of Rad-score (3 mm).


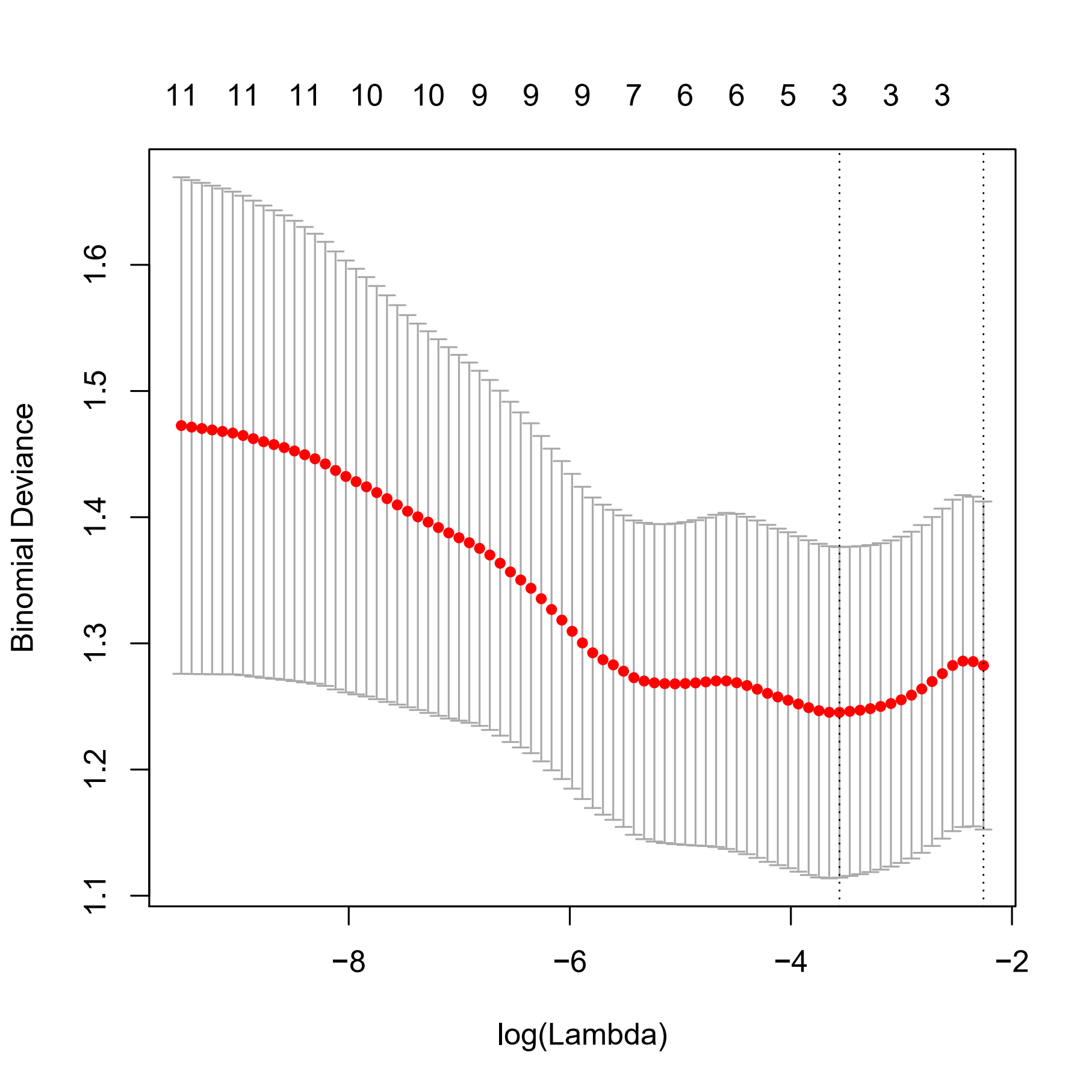

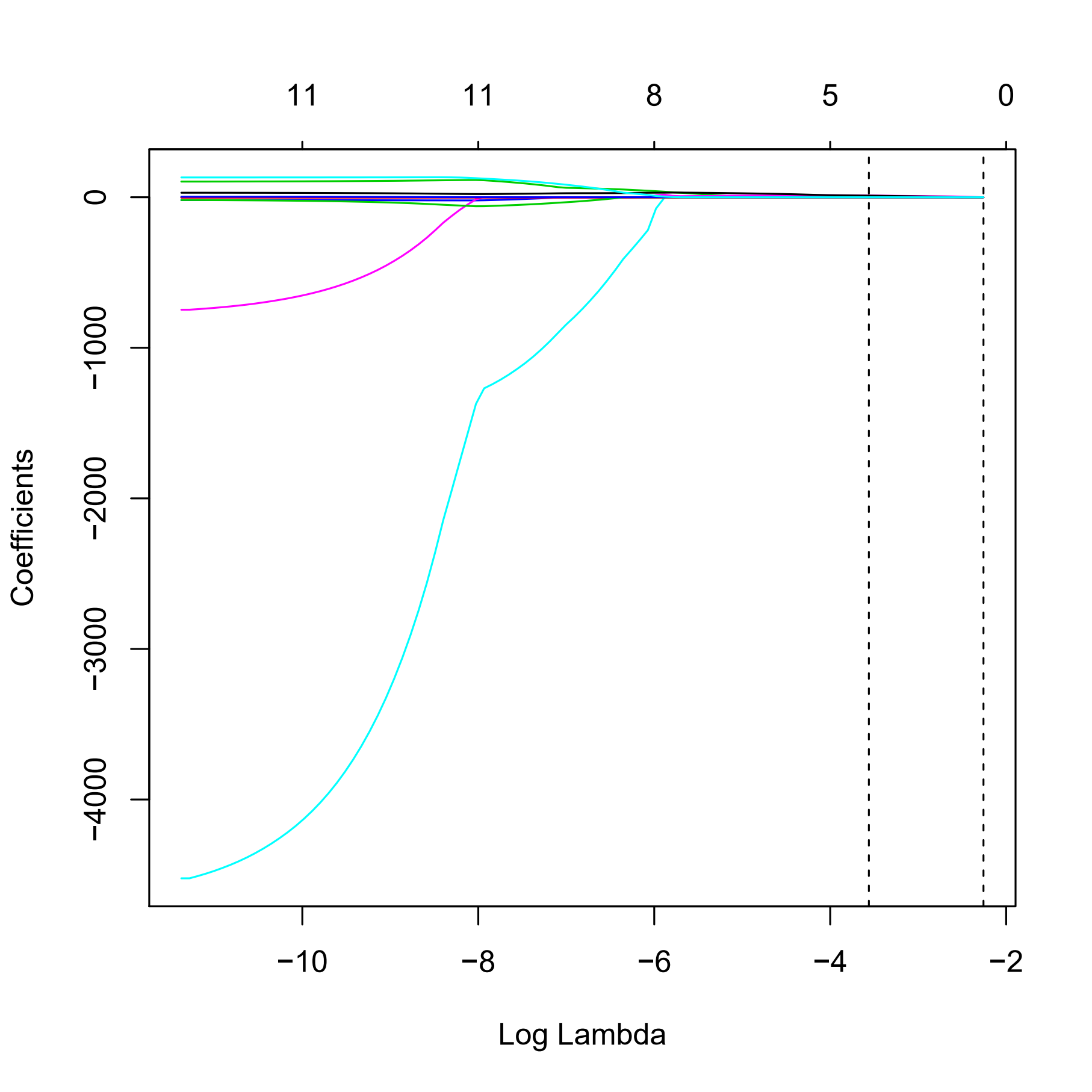

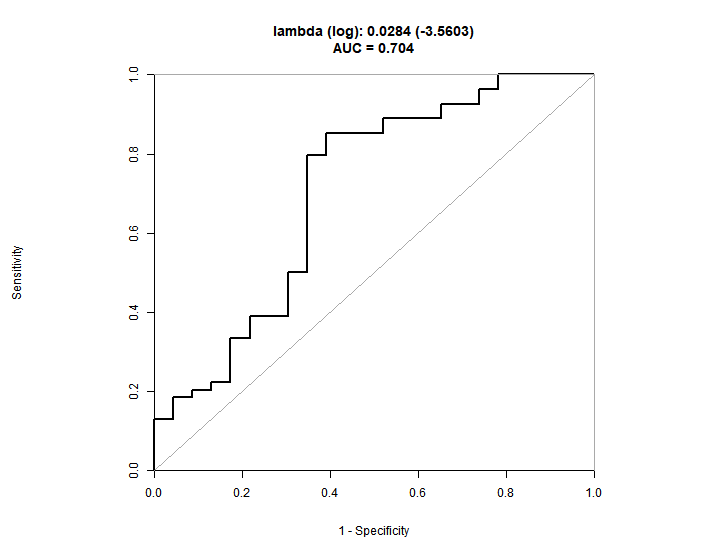


**Supplementary Figure S2.** LASSO algorithm and 10-fold cross-validation were carried out to extract the best subset of 1mm slice thickness CT radiomics features. (A) The best feature was selected based on the AUC value. The black vertical line represents the best λ value, and the model provides the best fit of the data. λ = 0.0284 with log（λ）= −3.5603 was selected as the best value. (B) LASSO coefficient curves for 12 radiomics features. The vertical line was the value selected by the 10-fold cross-validation in A, where the best λ results in 3 non-zero coefficients. (C) ROC curve of Rad-score (1mm).
